# Individual and food environment factors associated with the diet of disadvantaged adults in Flanders

**DOI:** 10.1093/eurpub/ckac130.104

**Published:** 2022-10-25

**Authors:** Y Inac, E de Clerq, N Van de Weghe, S Dury, S D'Hooghe, S Vandevijvere, D van Dyck, B Deforche, K De Ridder

**Affiliations:** 1 Department of Epidemiology and Public Health, Sciensano, Brussels, Belgium; 2 Department of Chemical and Physical Health Risks, Sciensano, Brussels, Belgium; 3 Department of Geography, Ghent University, Ghent, Belgium; 4 Department of Educational Sciences, Vrije Universiteit Brussel, Brussels, Belgium; 5 Department of Movement and Sports Sciences, Ghent University, Ghent, Belgium; 6 Department of Public Health and Primary Care, Ghent University, Ghent, Belgium

## Abstract

**Background:**

Health inequalities partially remain due to differences in diet between socioeconomic groups. Examining the association between socio-ecological factors and the diet of socioeconomically disadvantaged (SED) individuals can enhance the development of interventions to decrease health inequalities.

**Methods:**

In total, 278 SED adults residing in two Flemish municipalities completed a survey addressing sociodemographics, diet, health and their perceptions of the food environment. The objective food environment was examined by assessing food retailer information in street network-based buffers of 500m and 1000m around participants’ addresses. Linear regression was used to test assumptions.

**Results:**

Individual factors such as poor subjective health (OR0.58;CI 0.34-0.97), food insecurity (OR0.60;CI 0.38-0.94) and living alone (OR0.86;CI 0.75-0.98) were negatively associated with healthy dietary habits such as daily fruit and vegetable (FV) consumption. Positive perceptions on the availability of FV were positively associated (OR1.09;CI 1.02-1.17) with daily FV consumption. Objective food environmental factors showed a stronger association with unhealthy dietary habits. A greater amount of retailers within 1000m walking distance was negatively associated with fast-food (OR0.96;CI 0.94-0.99) and sugar-sweetened beverages (SSB) consumption (OR0.93; CI 0.88-0.98). More supermarkets within 500m distance was negatively associated (OR0.77;CI 0.58-0.97) with SSB consumption, while more convenience stores within a 1000m distance was positively associated (OR1.48;CI 1.17-1.88) with SSB consumption.

**Conclusions:**

Our findings suggest that factors associated with the diet of SED adults differ according to food and drink items. Interventions focused on this population should take these differences into account.

**Key messages:**

- Individual and food environmental factors are both associated with the diet of socioeconomically disadvantaged adults but differ according to food and drink items.
- Individual factors and perceptions of the food environment more likely associated with a healthy diet. Objective factors of the food environment were more likely associated with an unhealthy diet.

